# Lipoprotein glomerulopathy induced by ApoE Kyoto mutation in ApoE-deficient mice

**DOI:** 10.1186/s12967-021-02765-x

**Published:** 2021-03-04

**Authors:** Hongyan Wu, Jing Yang, Yun-Qiang Liu, Song Lei, Mei Yang, Zhi Yang, Yuan Yang, Zhangxue Hu

**Affiliations:** 1 Department of Nephrology and National Clinical Research Center for Geriatrics, West China Hospital, Sichuan University, Sichuan, Guoxue Alley, 37#, Wuhou District, Chengdu, 610041 China; 2 Department of Medical Genetics, West China Hospital, Sichuan University, Chengdu, China; 3 Department of Pathology, West China Hospital, Sichuan University, Chengdu, China

**Keywords:** Lipoprotein glomerulopathy, *ApoE Kyoto *(*p.R43C*), *ApoE Sendai *(*p.R163P*), Atherosclerosis, Recombinant adenovirus

## Abstract

**Background:**

Lipoprotein glomerulopathy (LPG) is a rare autosomal dominant kidney disease that is most commonly caused by mutations in ApoE Kyoto (p.R43C) and ApoE Sendai (p.R163P). Differences in phenotype among the various ApoE mutations have been suggested, but the pathogenic role of ApoE Kyoto has not been validated in an animal model. This study intended to establish an ApoE Kyoto murine model and to further compare the pathologic differences between ApoE Kyoto and ApoE Sendai.

**Method:**

Male ApoE-deficient mice, 3 months of age, were divided into five groups, including the AD-ApoE Sendai, AD-ApoE Kyoto, AD-ApoE3, AD-eGFP, and ApoE (−/−) groups. The first four groups received recombinant adenovirus that contained the entire coding regions of the human *ApoE Sendai* and *ApoE Kyoto*, *apoE3*, and *eGFP* genes, respectively. Fasting blood and urine samples were collected at multiple time points. Lipid profiles and urine albumin–creatinine ratio were measured. Renal and aortic histopathologic alterations were analyzed.

**Results:**

After virus injection, plasma human ApoE was detected and rapidly reached the maximum level at 4–6 days in the AD-ApoE Kyoto and AD-ApoE Sendai groups (17.4 ± 3.1 µg/mL vs.: 22.2 ± 4.5 µg/mL, respectively) and at 2 days in the AD-ApoE3 group (38.4 µg/mL). The serum total cholesterol decreased by 63%, 65%, and 73% in the AD-ApoE Kyoto, AD-ApoE Sendai and AD-ApoE3 groups, respectively. There were no significant changes in serum triglyceride and urinary albumin–creatinine ratio among the five groups. Typical lipoprotein thrombi with positive ApoE staining were detected in the AD-ApoE Kyoto and AD-ApoE Sendai groups. The Oil-red O-positive glomerular area tended to be higher in the AD-ApoE Kyoto group (9.2%) than in the AD-ApoE Sendai (3.9%), AD-ApoE3 (4.8%), AD-eGFP (2.9%), and ApoE (−/−) (3.6%) groups. The atherosclerotic plaque area in the aorta was lower in the group injected with various ApoE mutations than in the group without injection of ApoE mutation.

**Conclusions:**

In this animal study, we first established an ApoE Kyoto mutation murine model and confirmed its pathogenic role in LPG. Our results suggested that LPG may be more severe with the ApoE Kyoto than with the ApoE Sendai.

**Supplementary Information:**

The online version contains supplementary material available at 10.1186/s12967-021-02765-x.

## Background

Lipoprotein glomerulopathy (LPG) is a rare autosomal dominant kidney disease with incomplete penetrance. To date, more than 200 patients with LPG have been reported worldwide and were mainly of Japanese and Chinese origin [1]. The presence of massive lipoprotein thrombi containing ApoE in dilated glomerular papillary lumina is the typical pathologic manifestation [2, 3]. Nephrotic-range proteinuria, hypertriglyceridemia, and progressive renal dysfunction accompanied by elevated ApoE level are common among most patients, and half of the patients may eventually develop uremia in 1–27 years. It is interesting that cardiovascular events such as angina pectoris, myocardial infarction et al. were rarely reported in LPG patients despite abnormal lipid profiles. Renal pathological findings are crucial to diagnosis, since LPG patients with normal lipid profiles have been reported [4–6].

Pathogenesis of LPG involves ApoE mutation and dysfunction of macrophage although detailed mechanism remains unknown. Human ApoE is a 34-kDa protein with 299 amino acids and is encoded by the *ApoE* gene (*ApoE*), which is located at 19q13.2 and has four exons and three introns [7]. It mediates the tissue uptake of triglyceride-rich lipoproteins through both the low-density lipoprotein receptor (LDLR) and the LDL receptor-related protein pathways. Moreover, ApoE is a ligand for heparan sulfate proteoglycan. To date, 18 ApoE variants [1, 8] have been reported to be associated with LPG. *ApoE Sendai (p. Arg163Pro)*, which is located in the LDLR binding area, was the first reported ApoE mutation related with LPG in East Japan [9]. Yamamoto et al. proved the pathogenicity of *ApoE Sendai* by developing LPG in ApoE-deficient mice using virus-mediated transduction of *ApoE Sendai* [10]. *ApoE Kyoto *(*p. Arg43Cys*), which is another leading ApoE variant that is related to LPG, is located outside LDLR binding area and has been reported worldwide. Previously, we reported the clustering of LPG patients carrying the *ApoE Kyoto* in Sichuan province, China [2]; this has been the largest reported group of LPG until now. Matsunaga et al. showed that the binding ability of recombinant apoE3 Kyoto to LDLR was reduced to 10%, compared with that of recombinant apoE3, in vitro [11]. However, the role of ApoE Kyoto has not been validated in animal model. A recent study showed that patients with ApoE mutations in the LDLR binding area may have relatively high blood pressure and serum ApoE [12], suggesting phenotype differences among various ApoE mutations. In this study, we developed an LPG model in ApoE knockout mice by injecting adenovirus carrying ApoE variants, including *APOE Kyoto*, *APOE Sendai*, and *APOE3*, and further compared the laboratory and histopathologic differences among the LPG mice with various ApoE mutations.

## Methods

### Recombinant adenoviruses construction

Recombinant adenoviruses AD-apoE3, AD-ApoE Sendai, AD-ApoE Kyoto, and AD-eGFP containing the entire coding regions of human apoE3, ApoE Sendai, ApoE Kyoto, and enhanced green fluorescent protein were packaged by GeneChem Co., Ltd., (Shanghai, China). All transgene expressions were under the control of the immediate early promoter of cytomegalovirus. Viruses were purified by double cesium chloride gradient ultracentrifugation, and viral titer was determined by plaque assay and expressed as plaque-forming units (pfu). Purified virus aliquots were stored at − 80 °C.

### Vector validation in cultured HepG2 and 293 T cells

In order to estimate the adenoviral gene transfection and expression efficiency in vitro, HepG2 and 293 T cells were infected with four recombinant adenoviruses at multiplicity of infection (MOI) of 40 and 4, respectively. After incubation for 48 h, the GFP expression and infection efficiency were determined under fluorescence microscopy. The cell protein and growth medium were collected for Western blot and ELISA analyses. The protein of the transfected HepG2 and 293 T cells were extracted by a protein extraction kit (Bio Teke Corporation, China), and the protein concentration was determined by the BCA Protein Assay Kit (Bio Teke Corporation, China), according to the manufacturer’s protocol. Thereafter, total protein (15 µg) was loaded into each lane of acrylamide gel and subjected to SDS-PAGE. The membranes were incubated with antibodies against human ApoE (ab183597, Abcam) at 4 °C overnight; α-tubulin antibody (sc-8035, Santa Cruz Biotechnology) was used as an internal control to verify the basal expression level and equal protein loading. Target protein bands were captured by the chemiluminescence imager (iBright CL1000, Thermo Scientific).

### Reverse transcription quantitative polymerase chain reaction (RT-qPCR)

Total RNA was extracted using RNA extraction kit (Bio Teke Corporation, China), according to the manufacturer’s instructions. The reverse transcription of total RNA into cDNA was performed according to the instructions of HiScript®II Q RT SuperMix (R223-01, Vazyme, China). The TB Green™ Premix Ex Taq™ II kit (TaKaRa Bio, Dalian, China) was used for the qPCR. The results were performed using the CFX96^TM^real-time PCR detection system (Bio-Rad, USA), which had the following program design: denaturation step at 95 °C for 30 s, followed by 40 cycles of denaturation at 95 °C for 5 s, annealing at 62 °C for 30 secs, and extension at 72 °C for 30 s. The sequences of the primer pairs were as follows: *GAPDH-F*: ACGGATTTGGTCGTATTGGG; *GAPDH-R*: CGCTCCTGGAAGATGGTGAT; *ApoE-F*: CACAGGCAGGAAGATGAAGGTT; and *ApoE-R*: TAATCCCAAAAGCGACCCAGT. The relative amount of genes for internal control was calculated using the comparative 2^−∆∆CT^ method and was normalized to that of GAPDH.

### Animal model establishment

Male ApoE (−/−) mice on a C57BL/6 background were purchased from Beijing Vital River Laboratory Animal Technology Co., Ltd., and genotype was determined before experiment. All animals were bred and housed at the animal experiment center of Sichuan University and were maintained on normal chow diet (4% fat, 0% cholesterol). Twenty-seven weight- and age-matched (3 months of age) male ApoE (−/−) mice were randomly divided into five groups, as follows: AD-ApoE Sendai (n = 6), AD-ApoE Kyoto (n = 6), AD-ApoE3 (n = 6), AD-eGFP (n = 5), and ApoE (−/−) (n = 4). At multiple time points (baseline, and 2, 4, 6, 10, 14, 20, 34, 50, 65, 90 days after adenoviruses injection), blood was collected from the retroorbital venous plexus after a four-hour fasting. Plasma total cholesterol and triglyceride were detected by enzymatic assay kits (Wako Pure Chemical Co., Osaka, Japan). Accurate quantification of human ApoE in mouse plasma was determined by sandwich ELISA kit (Cat # EHAPOE, Thermo scientific). Urinary albumin was measured using a mouse albumin ELISA Quantification kit (Bethyl Laboratories, Montgomery, TX). Urine creatinine was determined by a creatinine assay kit (DICT-500, BioAssay System), according to the protocols of the manufacturer. The urine albumin–creatinine ratio (ACR) was calculated to estimate the amount of proteinuria.

### Histologic analysis

Kidneys were dissected after 90 days of virus injection. Renal sections were fixed in 4% paraformaldehyde, embedded in paraffin, and deparaffinized in xylene. Thereafter, the sections were stained with hematoxylin/eosin and periodic acid-Schiff. The rabbit antihuman ApoE polyclonal antibody (ab24139, Abcam) was used for immunofluorescence staining, which followed the standard protocol. Frozen sections were stained with Oil-red O. All the Oil-red O-positive glomeruli of five groups were captured, then the area was calculated by Image J software, the positive area ratio of each glomerulus and the percentage of positive glomeruli in five groups were analyzed finally. A small block of the kidney specimen was fixed with 2.5% glutaraldehyde for electron microscopy detection. Serial 5-µm-thick cryosections of the aortic valve were mounted on masked slides and stained with Oil-red O, the Oil-red O positive atherosclerotic plaque area and the area difference of aorta adventitia and intima in the aortic valve were quantified by the Motic images Advanced 3.2 software and the ratio of two parameters was described as atherosclerotic plaque area percentage.

### Statistical analysis

GraphPad prism 6 software was used to carry out statistical analyses, and all data were presented as mean ± SD. One-way analysis of variance test was used to compare the mean values between five groups, statistical significance for all comparisons was assigned at *p* < 0.05.

## Results

### Transfection efficiency and in vitro validation of recombinant adenovirus

**Fig. 1:**
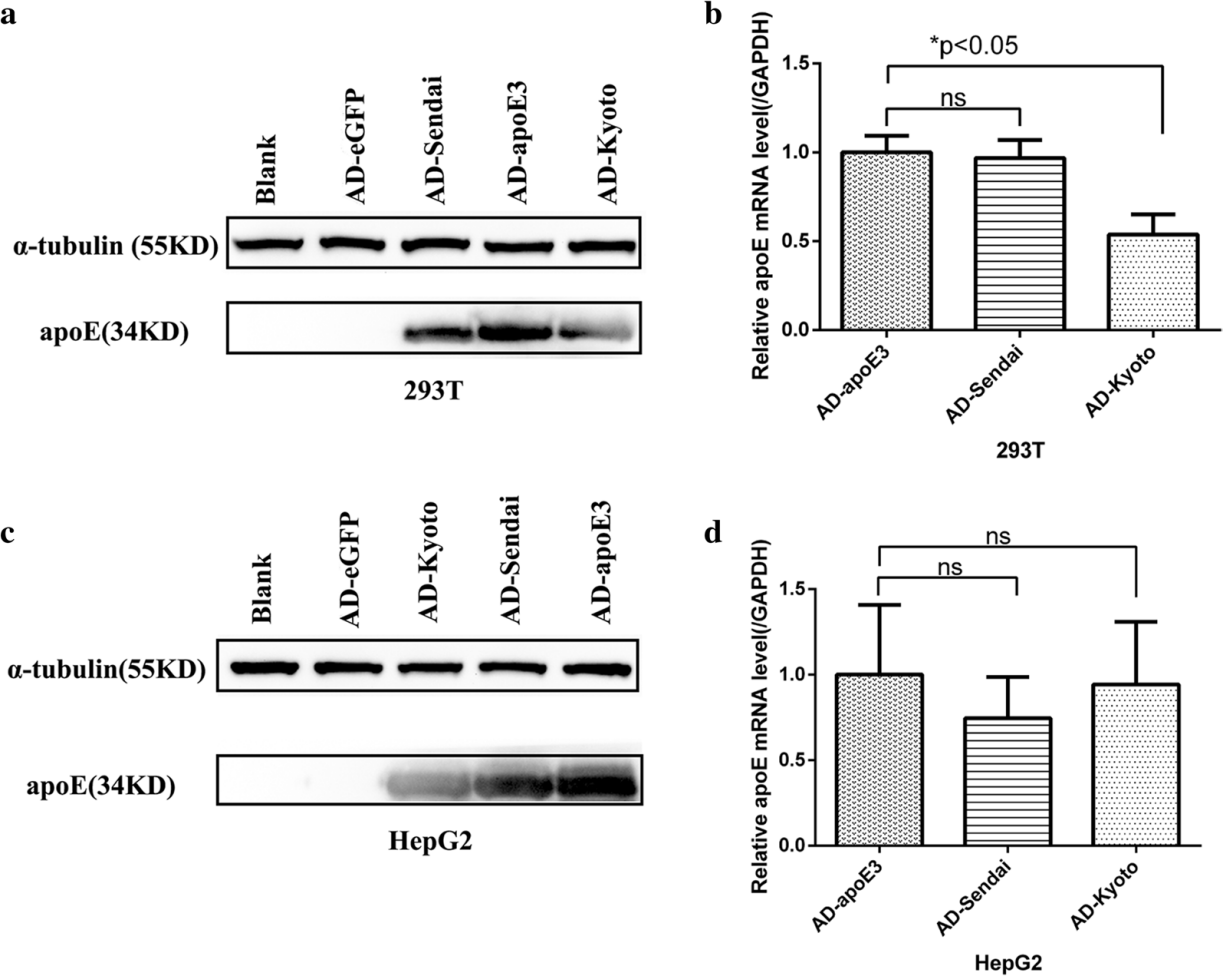
Overexpression of various human ApoE in 293 T and HepG2 cells induced by recombinant adenovirus transfection. Western blot and qPCR analysis show that human ApoE is overexpressed in the AD-apoE3, AD-ApoE Kyoto, and AD-ApoE Sendai groups but is absent in the AD-eGFP and blank groups

After transfecting the HepG2 and 293 T cells with four different recombinant adenoviruses, approximately 80% to 85% of the cells expressed eGFP at 48 h after infection. Western blot and qPCR analysis demonstrated that human ApoE was overexpressed in the cells infected with AD-apoE3, AD-ApoE Sendai, and AD-ApoE Kyoto but not in the cells infected with AD-eGFP (Fig. 1). In addition, the ELISA analysis of the cellular supernatant indicated that, at the same MOI, more human ApoE protein was secreted in the Ad-apoE3 group than in the other groups. On the other hand, no human ApoE was detected in the AD-eGFP group (Additional file 1: Table S1).

### Quantification of human ApoE in the plasma of ApoE (−/−) mice

**Fig. 2:**
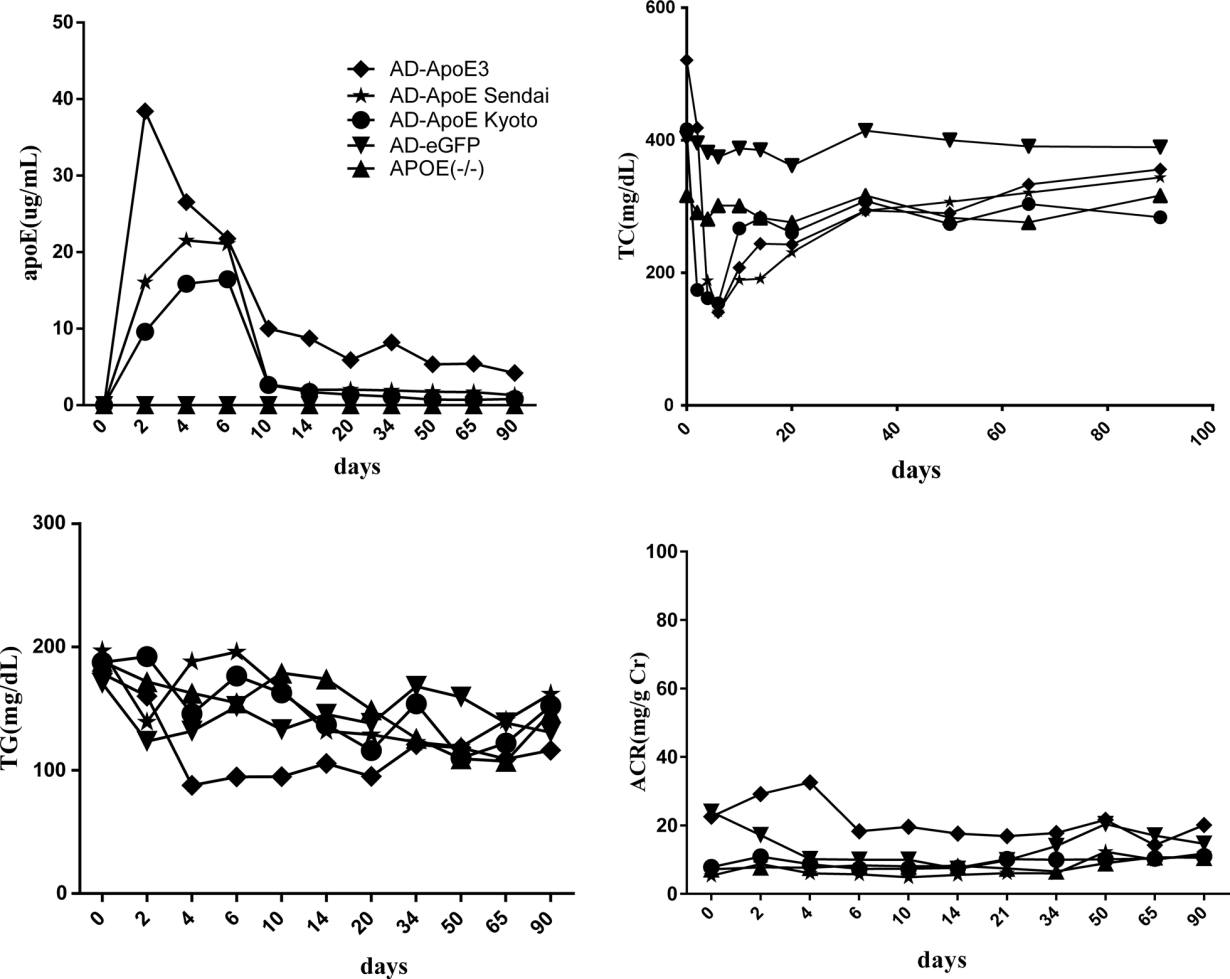
Alterations in the serum ApoE, total cholesterol (TC), total triglyceride (TG), and urinary albumin–creatinine ratio (ACR) in the five groups

In the AD-ApoE3 group, the expression of plasma human ApoE rapidly reached a peak of 38.4 µg/mL on the 2nd day after virus injection and declined to a low level on the 10th day. In the AD-ApoE Sendai and AD-ApoE Kyoto groups, human ApoE was detected on the 2nd day; reached the peak levels of 22.2 µg/mL and 17.4 µg/mL, respectively, after 4–6 days; and declined to the baseline level on the 10th day. There were no significant differences in the ApoE peak concentration between the AD-ApoE Kyoto and AD-ApoE Sendai groups. In addition, no ApoE was detected in the AD-eGFP and ApoE (−/−) groups (Fig. 2).

### Plasma total cholesterol and triglyceride and urine albumin–creatinine ratio detection

Among the normal chow-fed ApoE (−/−) mice, the mean baseline total cholesterol levels before virus injection were 416.7 ± 81.7 mg/dL in the AD-ApoE Kyoto group, 406.9 ± 29.1 mg/dL in the AD-ApoE Sendai group, 521 ± 50.6 mg/dL in the AD-ApoE3 group, 403.6 ± 50.9 mg/dL in the AD-eGFP group, and 316.8 ± 26.4 mg/dL in the ApoE (−/−) group. Along with the expression of human ApoE, plasma total cholesterol began to decrease to the lowest level at 4–6 days after virus injection and gradually increased to baseline thereafter. The relative reduction in total cholesterol level was 63%, 65%, and 73% in the AD-ApoE Kyoto, AD-ApoE Sendai, and AD-ApoE3 groups, respectively. There were no apparent changes in total cholesterol in the AD-eGFP and ApoE (−/−) groups.

Among the normal chow-fed ApoE (−/−) mice, the mean baseline triglyceride levels before virus injection were 187.7 ± 48.4 mg/dL in the AD-ApoE Kyoto group, 197 ± 41.8 mg/dL in the AD-ApoE Sendai group, 178.6 ± 31.4 mg/dL in the AD-apoE3 group, 170.7 ± 52.4 mg/dL in the AD-eGFP group, and 188.4 ± 37.5 mg/dL in the ApoE (–/–) group. Unlike the total cholesterol, the triglyceride and urine ACR levels had no apparent changes in the five groups during the observation period (Fig. 2).

The mean plasma total cholesterol and triglyceride in male wild mice on a C57BL/6 background from the same company were 95.39 ± 22.78 mg/dl, and 126.4 ± 25.2 mg/dl, respectively.

### Histologic manifestation of renal lesions

**Fig. 3:**
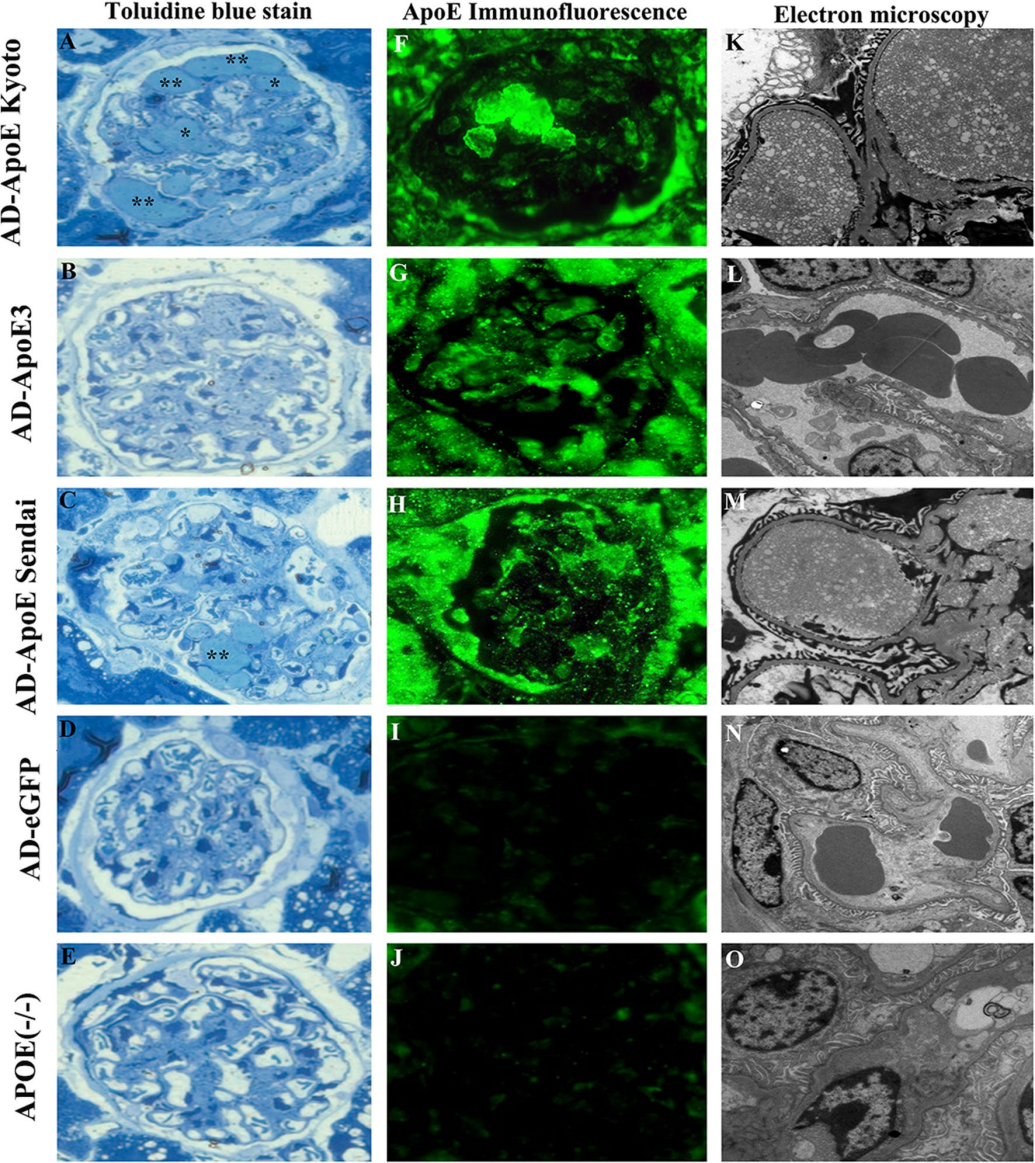
Renal pathologic lesions in the five groups. Panels **a**–**e** show the toluidine blue stain in the five groups. * represent the typical lipoprotein thrombi accumulated in the dilated capillary lumina of glomeruli in AD-ApoE Kyoto and AD-ApoE Sendai group. Panels **f**–**j** show ApoE immunofluorescence stain of glomeruli in five groups, positive ApoE staining are existed in AD-ApoE Kyoto, AD-ApoE3 and AD-ApoE Sendai groups. Panels **k**–**o** show electron microscopy manifestation of the five groups, osmiophilic granules and various lipid vacuoles accumulated in dilated capillary lumina and mesangial area of glomeruli in AD-ApoE Kyoto and AD-ApoE Sendai group but were absent in other groups

**Fig. 4:**
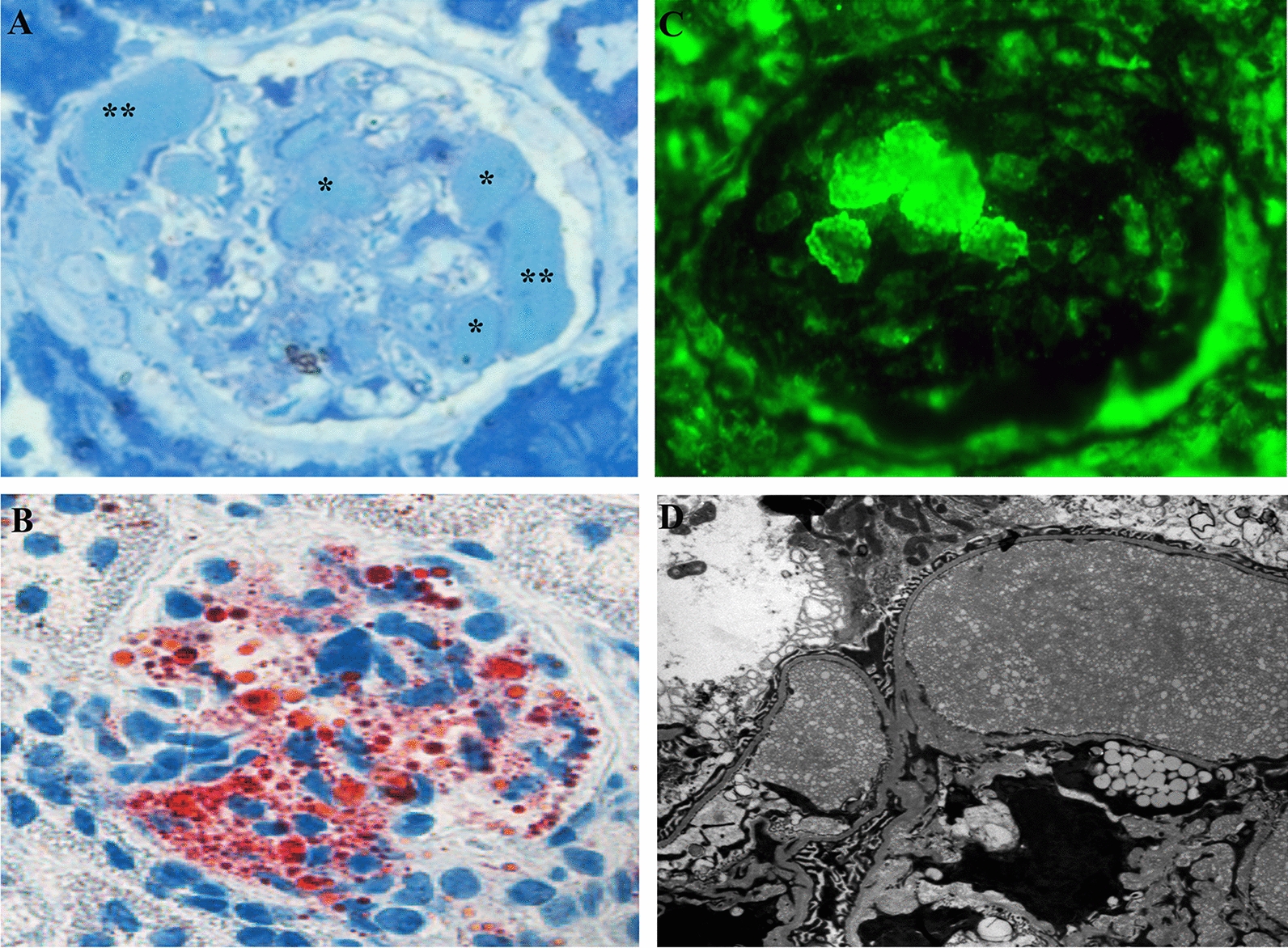
Typical renal pathologic lesions of lipoprotein glomerulopathy in the murine model. Panel **a** shows a glomerulus with accumulated lipoprotein thrombi in the dilated lumina of the glomerular capillaries (Toluidine blue staining, ×400), * show the lipoprotein thrombi accumulated area. Panel **b** shows ApoE primarily in the capillary lumina on immunofluorescence staining (frozen section, ×400). Panel **c** shows an obvious Oil-red O-positive area in the glomerulus (×400). Panel **d** shows numerous lipid granules deposited in the dilated capillary lumina of the glomeruli under electron microscope (×6000)

**Fig. 5:**
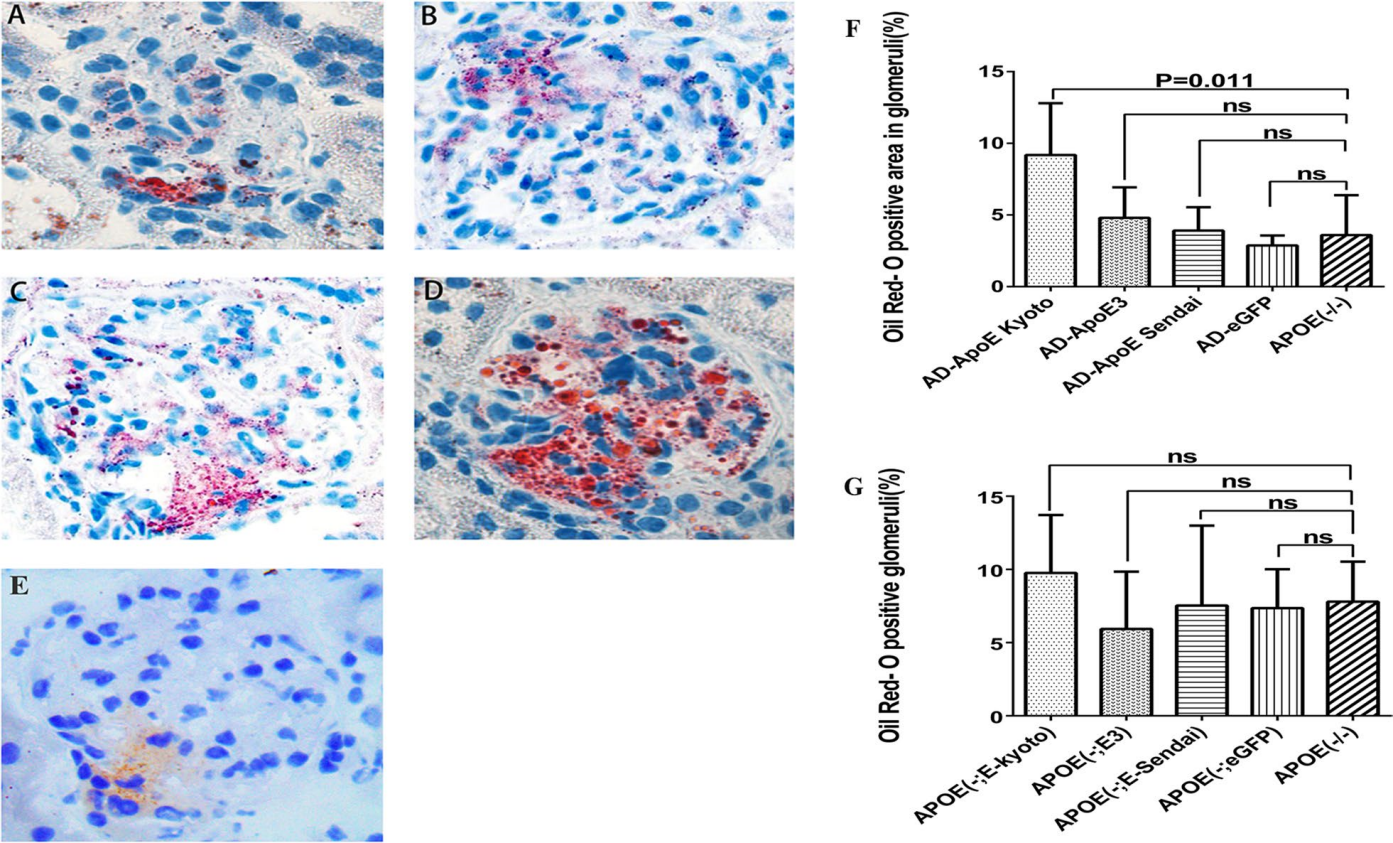
Oil-red O-positive staining and area analysis of the glomeruli in the five groups. Oil-red O-positive staining in the glomeruli are shown in (**a**) ApoE (−/−), **b** AD-eGFP, **c** AD-ApoE Sendai, **d** AD-ApoE Kyoto, and **e** AD-ApoE3. All the Oil-red O-positive glomeruli of five groups were captured. The Oil-red O-positive area was measured by Image J software. We calculated the positive area ratio of each glomerulus using the formula: positive area/total glomerular area, then averaged the ratio, which was shown in (**f**). The percentage of positive glomeruli was calculated by the formula: number of the positive glomeruli/number of total glomeruli, which was shown in (**g**)

The renal lesions in the five groups were estimated 90 days after adenovirus injection, as shown in Fig. 4. LPG-like renal lesions were observed in three mice of the AD-ApoE Kyoto group and in one mouse of the AD-ApoE Sendai group. Lipoprotein thrombi were observed in the dilated capillary lumina and mesangial area of the glomeruli by toluidine blue staining and were positive for ApoE and Oil-red O staining. Under electron microscopy, the thrombus-like substances comprised osmiophilic granules and lipid vacuoles of various sizes (Fig. 3). The typical renal lesions in the AD-ApoE Kyoto group are demonstrated in Fig. 4. There were no typical lipoprotein thrombi observed in the other three groups. The Oil-red O-positive glomerular area in AD-ApoE Kyoto group was 9.2% and tended to be higher, compared with that in the other groups [3.9% in AD-ApoE Sendai, 4.8% in AD-apoE3, 2.9% in AD-eGFP, and 3.6%, in ApoE (−/−) groups]. There was no difference in the Oil-red O-positive glomeruli percentage among the groups (Fig. 5).

### Analysis of atherosclerosis in the aortic valve

**Fig. 6:**
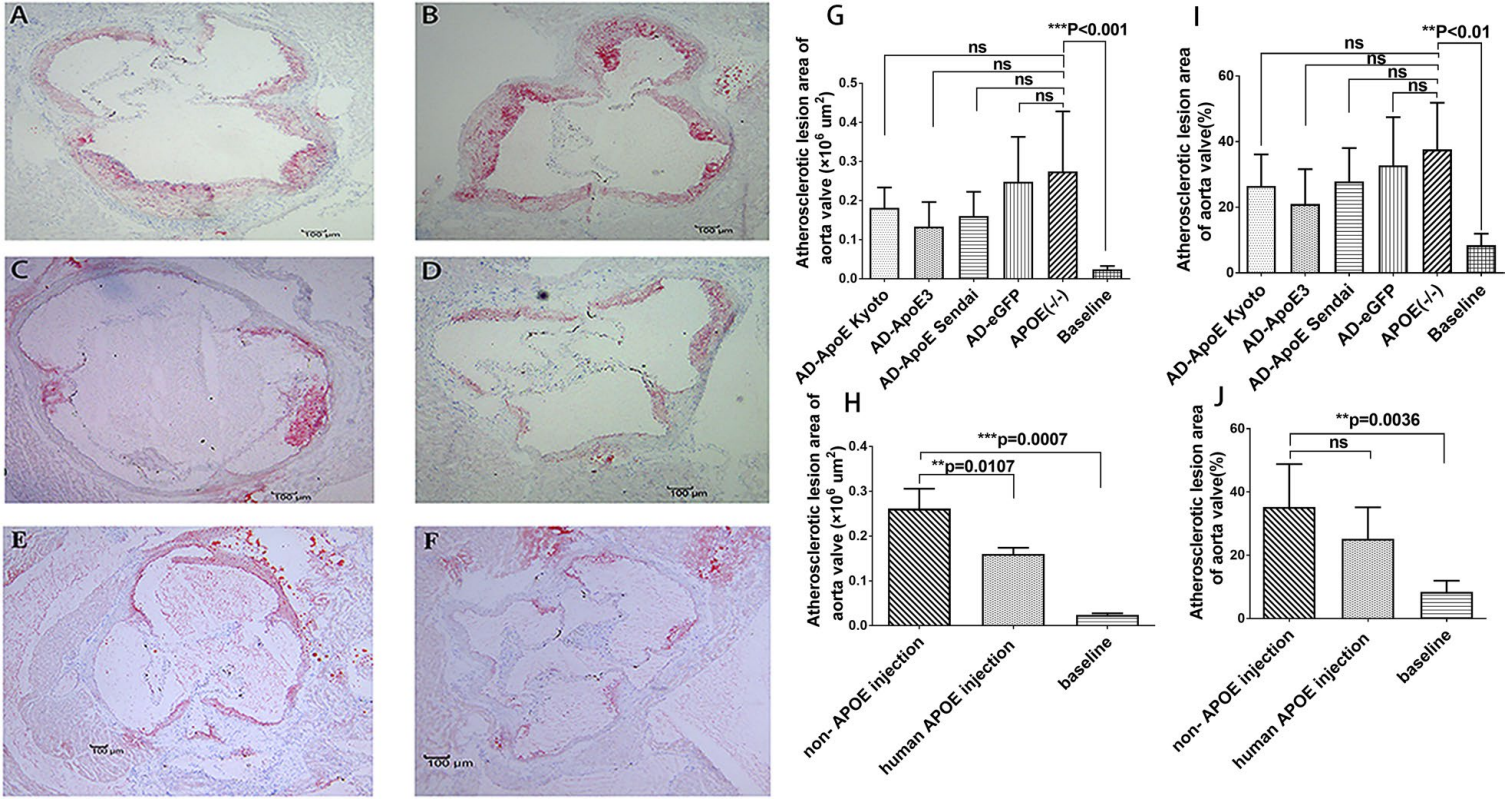
Oil-red O-positive staining and atherosclerosis plaque area in the aortic valve in the five groups. The atherosclerosis plaque with Oil-red O-positive staining in the aortic valve was observed in 3-month old ApoE (−/−) mice with chow diet and was shown in (**f**) as baseline group. Three month later, atherosclerosis plaque area of five groups were increased and shown in (**a**) ApoE (−/−), (**b**) AD-eGFP, (**c**) AD-ApoE Sendai, (**d**) AD-ApoE Kyoto, (**e**) AD-ApoE3. The atherosclerotic plaque area and its percentage in area difference of aorta adventitia and intima in the aortic valve were quantified by the Motic images Advanced 3.2 software, the (**g** and **h**) atherosclerosis plaque area and (**i** and **j**) percentage of aortic valve level are shown. In Panel (**h** and **j**), the AD-ApoE Sendai, AD-ApoE Kyoto, and AD-ApoE3 groups were pooled as human apoE injection group while the ApoE (−/−) and AD-eGFP groups were pooled as non-APOE injection group

Among the ApoE-deficient mice with normal chow diet, the mean atherosclerotic plaque area in the aortic root was 0.024 ± 0.008 × 10^6^ µm^2^. Three months later, the atherosclerotic plaque increased in size and measured 0.18 ± 0.05 × 10^6^ µm^2^, 0.16 ± 0.06 × 10^6^ µm^2^, 0.13 ± 0.06 × 10^6^ µm^2^, 0.25 ± 0.1 × 10^6^ µm^2^, and 0.27 ± 0.15 × 10^6^ µm^2^ in the AD-ApoE Kyoto, AD-ApoE Sendai, AD-apoE3, AD-eGFP, and ApoE (−/−) groups, respectively (Fig. 6). Atherosclerosis progression was slower in the three groups that were injected with various ApoE variants than in the other two groups.

Oil red O staining results in lung from AD-ApoE Sendai, AD-ApoE Kyoto and ApoE(−/−) groups. There are thrombi-like material in pulmonary capillaries. However, there are no differences among them (Additional file 2: Figure S1).

## Discussion

LPG is a rare inherited renal disease characterized by formation of intraglomerular lipoprotein thrombi secondary to lipid deposition in the lumina of severely dilated glomerular capillaries. The typical clinical manifestations of LPG include proteinuria or nephrotic syndrome and elevated serum ApoE. *ApoE Kyoto* has been the most common mutation in LPG. In this study, we first established an LPG murine model induced by ApoE Kyoto. Thrombi in dilated glomerular capillaries with positive staining for ApoE and Oil-red O were observed in the AD-ApoE Kyoto and AD-ApoE Sendai groups and were seen to comprise osmiophilic granules and lipid vacuoles of various sizes on electron microscopy. All of the above supported the pathogenicity of ApoE Kyoto in LPG.

Notably, the lipoprotein thrombi area seemed larger in the ApoE Kyoto mice than in the ApoE Sendai mice, although the lipid profiles of both groups were comparable. ApoE Kyoto and ApoE Sendai both have significantly reduced helical content and are thermodynamically destabilized and prone to aggregation [13]. ApoE Sendai is located in the LDLR binding area, and the affinity of ApoE Sendai for LDLR was decreased [10]. Recently, patients with LPG with mutations in the LDL receptor binding region were reported to have relatively high blood pressure and serum ApoE levels [12]. However, detailed pathologic analyses were lacking. Although ApoE Kyoto has reduced affinity for LDLR, it is located outside the LDLR binding area. Furthermore, triglyceride-rich lipoprotein containing ApoE Kyoto had been shown to have increased affinity for endothelial cells, and this may be manifested as local adhesion [14]. In our study, the lipoprotein thrombi seemed milder in the ApoE Sendai mice than in the ApoE Kyoto mice. Larger samples of LPG model and detailed clinicopathologic analysis of LPG are required to clarify this issue.

LPG is characterized by elevated serum ApoE and hypertriglyceridemia. In the study by Yamamoto, the expression of ApoE Sendai resulted in insufficient normalization of hypercholesterolemia in ApoE-deficient mice and induced high plasma levels of triglyceride [10]. The mechanism of plasma triglyceride induction remains unknown, but it was suggested to be related with the hepatic uptake of plasma cholesterol by the LDL receptor [10]. However, in our study, there was no pulse elevation of plasma triglyceride after virus injection, whereas serum ApoE increased accompanying with decreased total cholesterol concentration. Two weeks after injection, serum ApoE declined along with elevated cholesterol. In contrast to the LPG murine model that was established by Yamamoto et al. our model used younger experiment animal, lower adenovirus injection dosage, and longer observation period. Although we are uncertain if these conditions caused the differences, our work suggested that pulse elevation of plasma triglyceride may not be required for the development of an LPG murine model. In fact, patients with LPG and mild hyperlipidemia have been reported clinically [5, 15].

Although the ApoE Kyoto-induced murine LPG model has been founded, the rate of the affected glomeruli was fairly low. Many factors may have contributed to this result. First, adenovirus-mediated expression of ApoE variants can only last for 7–10 days and can immediately decrease thereafter; this was not consistent with the sustained expression of ApoE mutants in human LPG. Second, the function of macrophages in this model seemed intact. Ito et al. [16] showed that impairment of macrophage function resulting from FcRγ deficiency would facilitate the onset of LPG in the presence of the same ApoE abnormalities. Kanamaru et al. [17] presented that LPG-like lesions could be observed in FcRγ-deficient mice. Usually, in human LPG, foam-like macrophages in the glomeruli are rare. In this study, foam-like macrophages were observed and implied the intact function of macrophages. Third, normal fat chow may contribute to the low penetrance of LPG murine model. Defective function of apoE may hinder LDLR-mediated uptake and raise remnant lipoprotein concentration (of VLDL and chylomicrons), which is very sensitive to the diet. More fat chow may place the stress on the aggregation and thrombosis in the kidney. Moreover, the normal ACR of the ApoE variant groups implied that this murine model reflected an early stage of LPG.

Atherosclerosis has been rarely reported among patients with LPG [18], although dyslipidemia commonly persisted. A majority of animal experiments suggested that human apoE3 could postpone the progression of atherosclerosis by decreasing serum total cholesterol or changing the inflammatory cell composition of the local plaque [19–21]. In this study, we found that the atherosclerotic plaque area was lower in the ApoE mutant groups than in the ApoE-deficient mice, which is consistent with Tavori`s work [19]. The similarity of ApoE between human and mouse is about 71%. For mouse, human ApoE is a “mutated ApoE”, which may lose a part of function. However, the residual function of the “mutated ApoE” is capable of improving the lipid profiles and playing the protective role against atherosclerosis. It is noticeable that angina pectoris and myocardial infarction were rarely reported in LPG patients, which supports that hypothesis that the mutated ApoE still maintains anti-atherosclerotic effects although it causes LPG meanwhile based on its aggregation prone characteristics [22].

The limitation of this study is the numbers of animals are small. LPG is an autosomal dominant kidney disease with incomplete penetrance. The LPG murine model also showed incomplete penetrance. This makes statistical analysis difficult. It is especially difficult to compare the two pathogenic variants of apoE. Larger sample size of animal model was needed for further study.

## Conclusions

We validated the pathogenicity of ApoE Kyoto in LPG using an animal study. ApoE Kyoto may induce LPG while protecting against atherosclerosis. Further studies are required to clarify the different phenotypes among the ApoE variants.

## Supplementary Information

**Additional file 1: Table S1.** Human ApoE concentration in the cell medium after 48 h of transfection.

**Additional file 2: Figure S1.** Oil-red O staining in lung tissue among APOE(−/−) group, APOE(-/Sendai) and APOE(-/Kyoto) groups. Panel A、B、C showed oil red O staining results in lung from APOE(-/Sendai), APOE(-/Kyoto) and APOE(−/−) groups. There are thrombi-like material in pulmonary capillaries. However, there are no differences among them.

## Data Availability

The datasets used and/or analyzed during the current study are available from the corresponding author on reasonable request.

## Abbreviations

LPG: Lipoprotein glomerulopathy
ApoE: Apolipoprotein E
LDL: Low-density lipoprotein
LDLR: Low-density lipoprotein receptor
ACR: Albumin–creatinine ratio
