# Supplementary material for: Lipoprotein glomerulopathy induced by ApoE Kyoto mutation in ApoE-deficient mice
Source: J Transl Med. 2021 Mar 4;19:97. doi: 10.1186/s12967-021-02765-x (PMC7934380; doi:10.1186/s12967-021-02765-x)
Supplement: Supplementary file 1 — Additional file 1: Table S1. Human ApoE concentration in the cell medium after 48 h of transfection. [file 12967_2021_2765_MOESM1_ESM.docx]

**Table S1. Human ApoE concentration in the cell medium after 48 hours of transfection**

| Cell | ApoE Kyoto | ApoE Sendai | ApoE3 | AD-eGFP | MOI | Blank |
| --- | --- | --- | --- | --- | --- | --- |
| 293T | 0.03 µg/mL | 0.023 µg/mL | 0.28 µg/mL | 0 µg/mL | 4 | 0 µg/mL |
| HepG2 | 0.044 µg/mL | 0.008 µg/mL | 0.25 µg/mL | 0 µg/mL | 40 | 0 µg/mL |
