# Supplementary material for: Lipoprotein glomerulopathy induced by ApoE Kyoto mutation in ApoE-deficient mice
Source: J Transl Med. 2021 Mar 4;19:97. doi: 10.1186/s12967-021-02765-x (PMC7934380; doi:10.1186/s12967-021-02765-x)
Supplement: Supplementary file 2 — Additional file 2: Figure S1. Oil-red O staining in lung tissue among APOE(−/−) group, APOE(-/Sendai) and APOE(-/Kyoto) groups. Panel A、B、C showed oil red O staining results in lung from APOE(-/Sendai), APOE(-/Kyoto) and APOE(−/−) groups. There are thrombi-like material in pulmonary capillaries. However, there are no differences among them. [file 12967_2021_2765_MOESM2_ESM.docx]

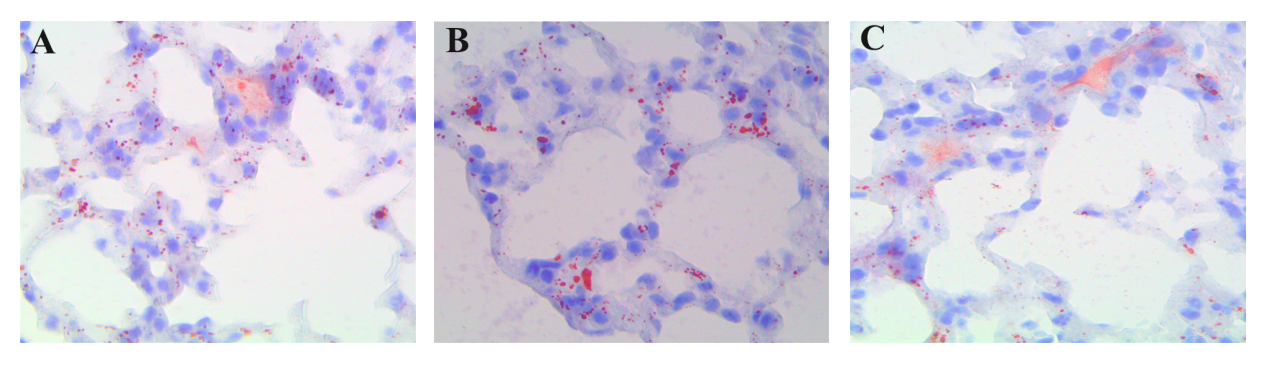


**Figure S1. Oil-red O staining in lung tissue among APOE(-/-) group, APOE(-/Sendai) and APOE(-/Kyoto) groups**

Panel A、B、C showed oil red O staining results in lung from APOE(-/Sendai), APOE(-/Kyoto) and APOE(-/-) groups. There are thrombi-like material in pulmonary capillaries. However, there are no differences among them.
